# Quantitative proteomics reveals significant variation in host responses of cattle with differing buffalo fly susceptibility

**DOI:** 10.3389/fimmu.2024.1402123

**Published:** 2024-07-16

**Authors:** Muhammad Kamran, Ali Raza, Muhammad N. Naseem, Conny Turni, Ala E. Tabor, Peter James

**Affiliations:** ^1^ The University of Queensland, Queensland Alliance for Agriculture & Food Innovation, Centre for Animal Science, St Lucia, QLD, Australia; ^2^ University of Copenhagen, Faculty of Health and Medical Sciences, Department of Veterinary and Animal Sciences, Frederiksberg, Denmark; ^3^ The University of Queensland, School of Veterinary Science, Gatton, QLD, Australia; ^4^ The University of Queensland, School of Chemistry and Molecular Bioscience, Brisbane, QLD, Australia

**Keywords:** immune response, cattle, host resistance, proteomics, *Haematobia irritans exigua*, buffalo fly, biomarkers

## Abstract

**Background:**

Control of buffalo flies (*Haematobia irritans exigua*, BFs) relies mainly on chemical methods; however, resistance to insecticides is widespread in BF populations. Breeding for resistance to BFs represents a possible alternative, but direct phenotyping of animals is laborious and often inaccurate. The availability of reliable diagnostic biomarker(s) to identify low BF carrier cattle would facilitate rapid and accurate selection for genetic improvement. However, limited information is available regarding differences amongst cattle in host responses to BF infestation.

**Methods:**

This study investigated the variation in Brangus cattle serum proteomic profiles before (naïve) and after peak BF exposure, in low (LF) and high BF burden (HF) cattle. Cattle were phenotyped for susceptibility based on BF counts on multiple dates using visual and photographic techniques. The relative abundance of serum proteins in cattle before and after exposure to BFs was analysed using sequential window acquisition of all theoretical fragment ion mass spectrometry (SWATH-MS).

**Results:**

Exposure to BFs elicited similar responses in HF and LF cattle, with 79 and 70 proteins, respectively, showing significantly different abundances post exposure as compared to their relevant naïve groups. The comparison of serum samples from naïve HF and LF cattle identified 44 significantly differentially abundant (DA) proteins, while 37 significantly DA proteins were identified from the comparison between HF and LF cattle post-exposure to BFs. Proteins with higher abundance in naïve LF cattle were enriched in blood coagulation mechanisms that were sustained after exposure to BFs. Strong immune response mechanisms were also identified in naïve LF cattle, whereas these responses developed in HF cattle only after exposure to BF. High BF cattle also showed active anticoagulation mechanisms in response to BF exposure, including downregulation of coagulation factor IX and upregulation of antithrombin-III, which might facilitate BF feeding.

**Conclusion:**

Underlying differences in the abundance of proteins related to blood coagulation and immune response pathways could potentially provide indirect indicators of susceptibility to BF infestation and biomarkers for selecting more BF-resistant cattle.

## 1 Introduction

The Australian cattle industry loses AUD$170.3 million annually due to buffalo fly (*Haematobia irritans exigua*) infestation (1), whereas costs of closely related horn flies (*Haematobia irritans irritans*) have been estimated at US$1 billion (2) and US$2.54 billion (3) in the United States and Brazil, respectively. Buffalo flies (BF) take a blood meal up to 40 times a day, causing cutaneous lesions (4), reduced milk yield and reduced weight gain (5). Control of BFs and horn flies has been shown to result in improved growth rates of up to 15-18% (6, 7) and increased feed efficiency (8). Currently, control relies mainly on the use of insecticides (9, 10), but widespread resistance to insecticides (11, 12) and increasing demand for chemical free animal products requires the development of more sustainable control strategies.

It has been observed that the magnitude of BF burdens varies between cattle both within and between breeds (13) and similar variation is observed with horn flies (14, 15). Feeding by *Haematobia* spp. stimulates several different immunological responses to salivary antigens (16, 17) and variation in fly burden has been associated with natural innate and acquired resistance (16). However, although, cattle exposed to BFs developed serum antibodies to fly antigens at levels that were correlated with the intensity of BF infestation, these antibodies were not protective (17). Exposure of animals to other hematophagous arthropod ectoparasites, such as ticks, mosquitoes, and other flies, has also been shown to evoke an immune response to salivary antigens (18, 19). Differential antibody responses against *Anopheles gambiae* salivary antigens in humans naturally exposed to these mosquitoes have been used as biomarkers to determine host exposure to bites (20).

Response to horn flies has been shown to affect blood consumption of the flies and the level of infestation (16). When the horn fly load exceeded 200 per animal, a reduction of blood intake and fly load was observed three weeks later and was associated with a decline in horn fly numbers. Antibody responses in all cattle diminished as fly numbers declined. This suggests that characterising differences in protective responses of high and low BF burden cattle may help identify biomarkers or immunological indicators to facilitate breeding for BF resistance. In this study we used sequential window acquisition of all theoretical fragment ion mass spectrometry (SWATH-MS) to investigate variation in abundance of serum proteins in Brangus (3/8 Brahman and 5/8 Angus) cattle with low and high BF burdens, before and after exposure to BFs.

## 2 Materials and methods

### 2.1 Animals and phenotyping for BF burden

Thirty-five two-year old Brangus steers were kept at Pinjarra Hills research station (27°32’12.3”S 152°55’19.1”E; University of Queensland Animal Ethics approval No. QAAFI469/18) and phenotyped as resistant, with low fly burden (LF) and susceptible with high fly burden (HF) using visual and camera techniques. The steers were sourced from a BF-free region in Australia and had no previous exposure to BFs. Buffalo fly numbers were estimated on one side of each animal by visual scoring and photography with a DSLR (Canon SX40 HS Powershot) digital camera, with 35X optical zoom while the cattle were held in a small paddock on 7 dates; 24 January, 27 February, 12 March, 26 March, 9 April, 28 April, 7 May, 9 October in 2020 and 20 January 2021. Seven cattle were classified as LF, and seven were classified as HF based on overall counts.

### 2.2 Sample collection

Samples were collected before exposure to BFs (timepoint-0) and post exposure to BFs during peak BF season (timepoint-1). Blood samples were collected by jugular venepuncture or tail bleeding with animals restrained in a crush. Sera were harvested from blood samples collected into 1×9 mL Vacuette^®^ Z clot activator tubes and stored at -20°C until further use. Samples collected from LF and HF groups at the two time points were used for quantitative proteomics.

### 2.3 Filter-aided sample preparation

Protein concentration of each serum sample was measured with Qubit^®^ protein assay (Thermo Fisher Scientific^®^, USA). Pierce concentrator 10K molecular weight cut-off (MWCO) columns (Thermo Fisher Scientific^®^, USA) were used for denaturation, reduction and alkylation of serum samples (21). For each sample, approximately, 150 µg of protein was denatured with 100 µL of 8 M urea and 50 mM ammonium bicarbonate (ABC). The denatured samples were loaded onto the 10K MWCO columns, which were then centrifuged at 14000g at room temperature for 40 minutes to allow the solution to pass through the membrane with 20 µL remaining on the top of the column. The wash solution (200 µL of 8 M urea and 50 mM ABC) was added followed by centrifugation at 14000 x g for 40 minutes at room temperature, and the filtrate was discarded. Reduction of proteins was accomplished by adding 200 µL of wash buffer containing 5 mM dl-Dithiothreitol (Sigma-Aldrich^®^, USA) with 30 minutes incubation at 56°C. Alkylation of cysteines was carried out in the dark with iodoacetamide (IAA) at a final concentration of 25 mM for 30 min incubation at room temperature. Dl-Dithiothreitol was added at 5 mM final concentration to quench the remaining IAA. The samples were then digested with 6 µg trypsin (Proteomics grade, Promega^®^, USA) in 100 µL of 50 mM ABC with overnight incubation at 37°C in a thermomixer (Eppendorf Thermomixer^®^ C, Germany) at 400 rpm. The digested peptides were collected by centrifugation followed by rinsing the membrane with 0.5 M NaCl (50 µL) and centrifugation. After combining the two filtrates, C18 ZipTips (Millipore^®^, USA) were used for the desalting of trypsin digested peptides according to the manufacturer’s recommendations. Before desalting, 5 µL of each sample was taken to generate a pooled sample containing almost 140 µg peptides. Fractionation of the pooled sample was carried out using Pierce High pH Reversed-phase Peptide Fractionation kit (Thermo Fisher Scientific^®^, USA). After loading the peptides onto the column containing 20 mg of resins in a 1:1 water/DMSO slurry, peptides were washed with 500 μL of LC-MS Grade water (Thermo Fisher Scientific^®^, USA) followed by elution in eight separate fractions of acetonitrile (300 μL for each 5%, 7.5%, 10%, 12.5% 15%, 17.5%, 20% and 50%) in triethylamine (0.1%). After drying in a vacuum concentrator (Eppendorf, Concentrator Plus, UK), the eluted peptides were resuspended in 0.1% trifluoroacetic acid.

### 2.4 Mass spectrometry

LC-MS/MS (undertaken by UQ’s School of Chemistry and Molecular Biosciences Mass Spectrometry facility) was used to measure the peptides using a Shimadzu^®^ Prominence nanoLC system with a TripleTOF 5600 mass spectrometer equipped with a Nanospray III interface (SCIEX^®^) (22). Peptides (2 µg) were desalted for 3 minutes at a 30 µL/min flow rate on an Agilent C18 trap (pore size = 300 Å, 0.3 mm i.d. × 5 mm, particle size = 5 μm) and separated at 1µL/min flow rate on a Vydac EVEREST reverse phased C18 HPLC column (pore size = 300 Å, 150 μm i.d. × 150 mm, particle size = 5 μm). Separation of peptides was completed over 45 minutes using buffers A and B with 1% acetonitrile/0.1% formic acid and 80% acetonitrile/0.1% formic acid ratios, respectively. Voltage and gas adjustments were according to the requirements. For data-dependent acquisition (DDA), MS-TOF scan was performed for 0.5 seconds across 350-1800 *m/z*, followed by DDA MS/MS of the top 20 peptides with automated selection at an intensity greater than 100 using 40 ± 15 V collision energy across 40-1800 *m/z* (0.05 seconds per spectrum). Data-independent acquisition (DIA) SWATH analyses were performed with MS scan followed by high sensitivity DIA mode scan across 350-1800 *m/z* and 26 *m/z* isolation windows, for 0.05 seconds and 0.1 seconds, respectively. The Analyst software automatically assigned the collision energy values for SWATH samples based on *m/z* mass windows (SCIEX^®^).

### 2.5 Data analysis

ProteinPilot software (SCIEX^®^5.0.2) was used to identify proteins from the DDA data, searching against all bovine proteins in UniProtKB database (downloaded 11 May 2021; 46754 total entries). The settings for ID search were as follows: sample type = identification, ID focus = biological modifications, instrument = TripleToF5600, species = none, cysteine alkylation = iodoacetamide, digestion = trypsin and search effort = thorough ID. The SWATH data was analysed using an ion library of proteins identified by ProteinPilot with a false discovery rate of 1%. Peptide abundance of each sample was measured by PeakView 2.1 (SCIEX^®^, USA) using the following settings: shared peptides = allowed, XIC extraction widow = 6 minutes, peptide confidence threshold = 99%, XIC width = 75ppm and FDR=1%. The MS proteomics data was submitted to the ProteomeXchange Consortium via the PRIDE (23) partner repository with the dataset identifier PXD039655. Statistical analyses were performed as reported by Kerr et al. (24) in R using ReformatMS and MsStats (25) with Benjamini and Hochberg corrections with a significance threshold of P < 10^-5^ for multiple comparison adjustments. A cut-off value of > 0.1 for log_2_ fold change (FC) was used as inclusion criteria of proteins for further analysis. For the identification of protein-protein interactions, Search Tool for the Retrieval of Interesting Genes/Proteins (STRING) (Version 11.5, https://string-db.org/) was used. It also provided enrichment analyses of gene ontology (GO) terms for biological processes (BP) using a target list consisting of UniProt accession identifiers (IDs) of significantly differentially abundant proteins (26). The STRING analysis used the *Bos taurus* genome (*Bos taurus* assembly ARS-UCD1.3) as background with the following settings: active sources including databases, co-occurrence, experiments, gene expression, co-expression and neighbourhood; meaning of network edges as evidence; high confidence (0.700) for the minimum required interaction score and 3 cluster sets of *k*-means clustering.

## 3 Results

### 3.1 Protein identification

Protein Pilot software (SCIEX^®^5.02) search identified a total of 206 proteins in the ion library (**Supplementary File 1**: **Supplementary Table S1**). SWATH-MS using PeakView 2.1 (SCIEX^®^) identified 136 proteins at 1% FDR cut-off (**Supplementary File 1**; **Supplementary Table S2**). Comparison of serum proteins from HF and LF cattle following infestation with their corresponding naïve samples (HF-0 vs HF-1 and LF-0 vs LF-1) indicated the response of each group to BF infestation. Differences between high and low fly cattle were determined by comparing samples before BF infestation (HF-0 vs LF-0) and during the peak BF season (HF-1 vs LF-1).

### 3.2 Response of cattle to BF exposure

**Figure 1:**
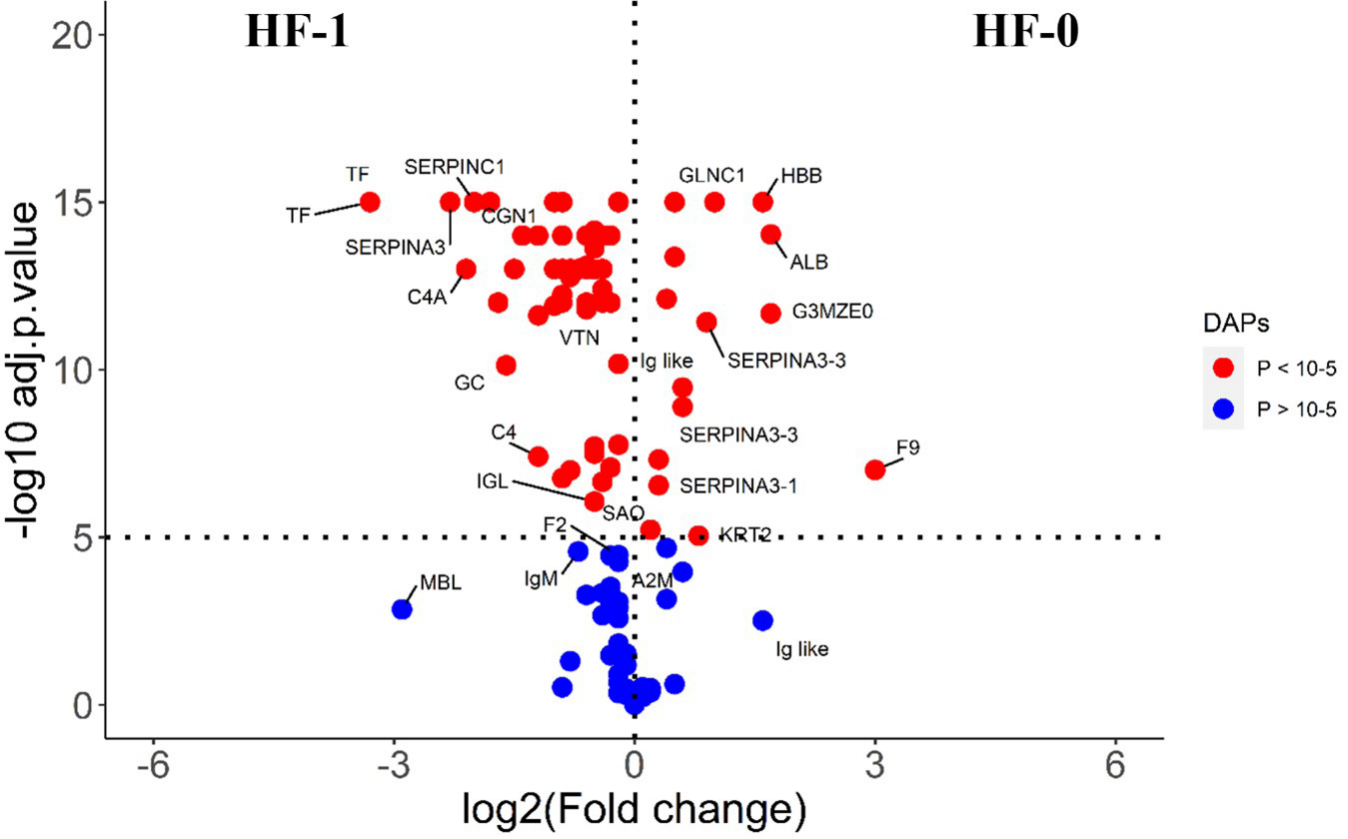
Volcano plot illustrating the DA proteins in high fly burden cattle in response to buffalo fly exposure (HF-0 vs HF-1). Red, significantly different in abundance (P < 10^-5^). Blue, not significantly different in abundance (P > 10^-5^) using cut-off value of > 0.1 for log_2_ fold change (FC) as inclusion criteria of proteins. Positive scale of X-axis indicates HF-0 (naïve, high fly burden) cattle and negative scale represent HF-1 (post exposure, high fly burden) cattle.

**Figure 2:**
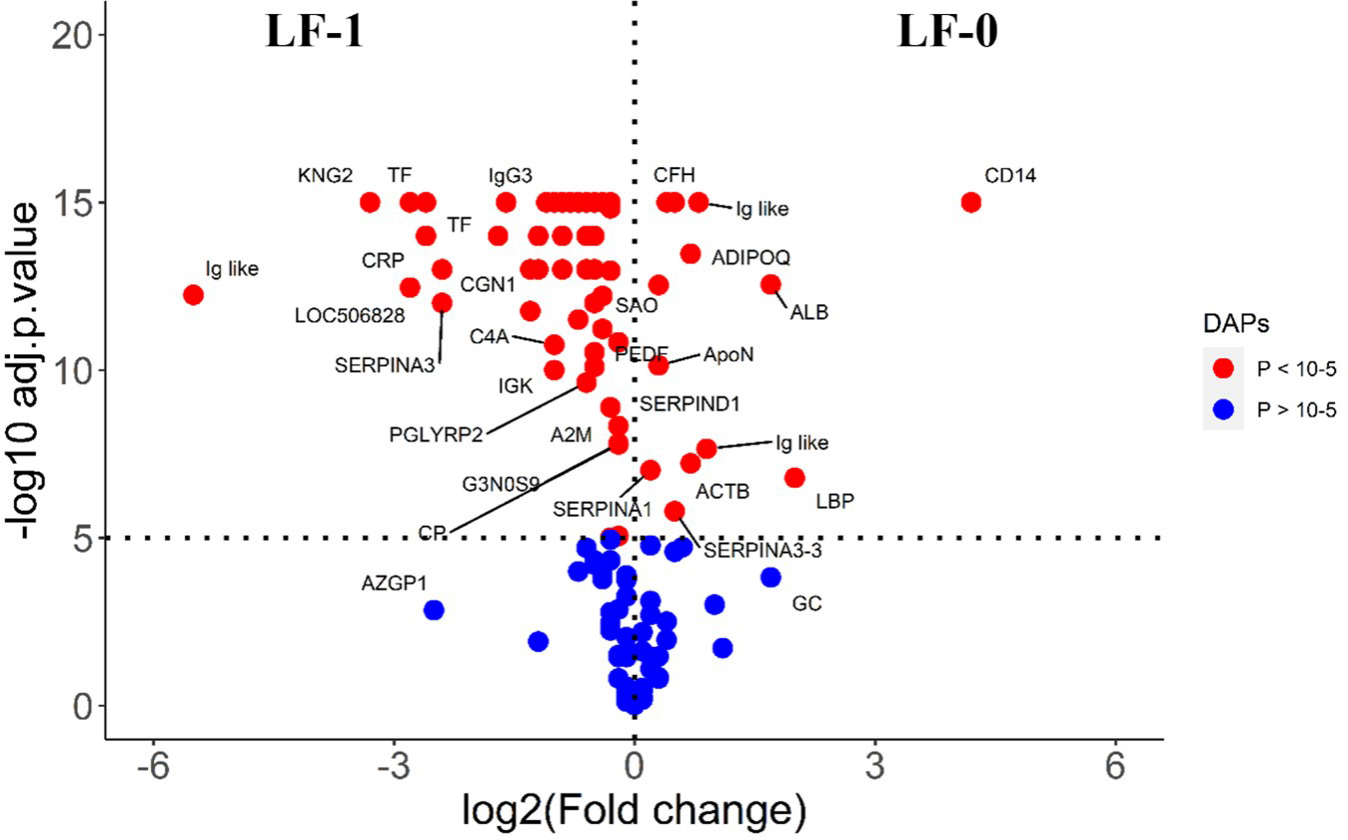
Volcano plot illustrating the DA proteins in low fly burden cattle in response to buffalo fly exposure (LF-0 vs LF-1). Red, significantly different in abundance (P < 10^−5^). Blue, not significantly different in abundance (P > 10^−5^) using cut-off value of > 0.1 for log_2_ fold change (FC) as inclusion criteria of proteins. Positive scale of X-axis indicates LF-0 (naïve, low fly burden cattle) and negative scale represent LF-1 (post exposure, low fly burden) cattle.

**Figure 3:**
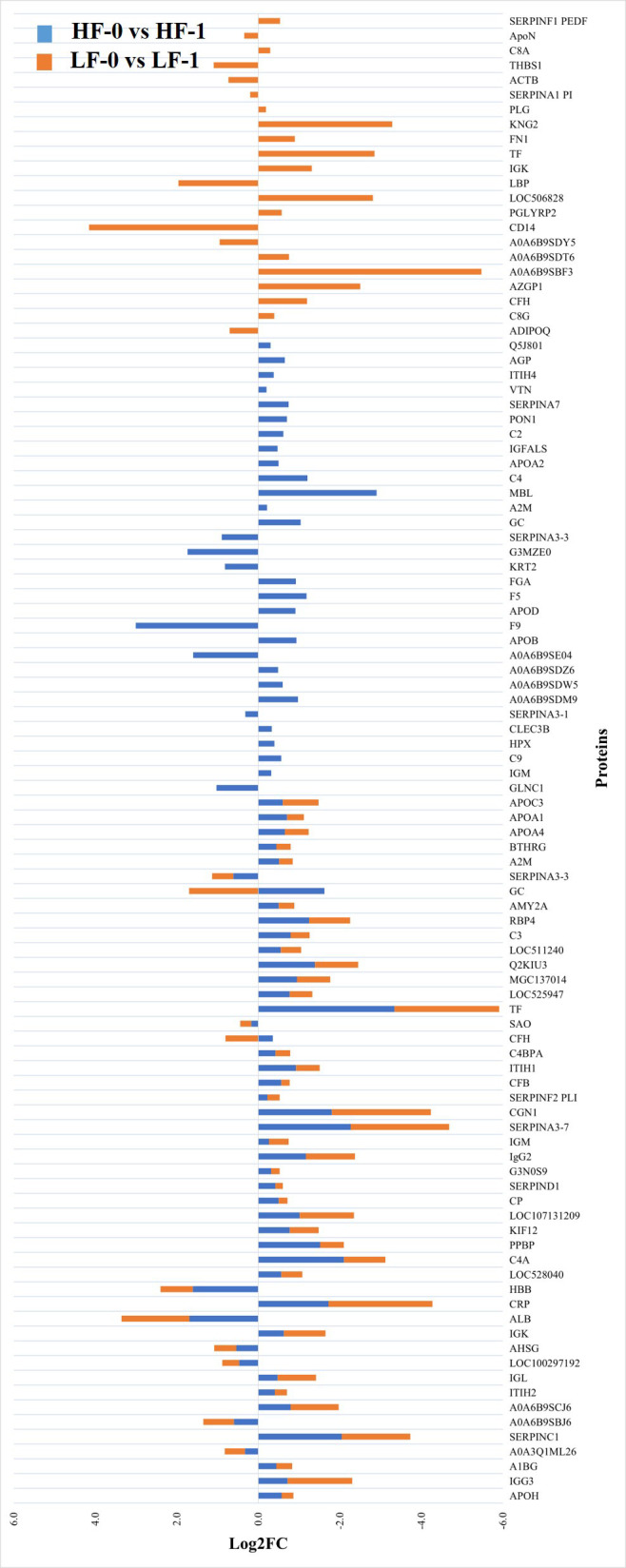
Relative abundance (log_2_ fold change (log_2_FC)) of significantly differentially abundant common and unique proteins between high fly burden (HF-0 vs HF-1) indicated with blue colour bars and low fly burden (LF-0 vs LF-1) cattle indicated with orange colour bars in response to BF exposure. Unique proteins in both comparisons are shown with single colour (either orange or blue) bars and common proteins are shown with double colour bars (orange and blue). A cut-off value of > 0.1 for log2 fold change (FC) is used as inclusion criteria of proteins.

The comparison of post-exposure HF cattle serum samples with the HF naïve samples (HF-0 vs HF-1) identified 79 DA proteins, with 64 proteins showing significantly higher abundances in response to BF infestation (**Figure 1**; **Supplementary File 1**: **Supplementary Table S3**). In LF cattle, LF-0 vs LF-1 comparison identified 70 DA proteins, with 52 proteins showing significantly higher abundance in response to BF infestation (**Figure 2**; **Supplementary File 1**: **Supplementary Table S4**). There were 48 proteins common in both comparisons, of which 38 and 40 proteins showed increased abundance in LF and HF cattle, respectively. The abundances of apolipoproteins A-IV (V6F7X3; APOA4), complement factor B (P81187; CFB), antithrombin-III (A0A3Q1NJR8; SERPINC1), complement C3 (Q2UVX4; C3), complement component 4A (E1BH06; C4A), C-X-C motif chemokine (F1MD83; PPBP), serotransferrin (Q29443; TF) and conglutinin (P23805; CGN1) were increased in both groups (**Figure 3**). Immunoglobulin-like proteins (5 in HF and 3 in LF) were also common between the two comparisons; however, these were identified with different UniProt accession IDs. The abundance of vitamin D binding protein (Q3MHN5; GC) and complement factor H (Q28085; CFH) was reduced in LF cattle and increased in HF cattle in response to BF exposure. Among common proteins, alpha-2-HS-glycoprotein (B0JYN6; AHSG) and albumin (B0JYQ0; ALB) were reduced in abundance in LF cattle after exposure to BFs. There were 31 and 22 uniquely DA proteins in HF and LF cattle comparisons, respectively (**Figure 3**). Of 31 uniquely abundant proteins in HF cattle, 24 proteins showed increased abundance after exposure to BFs, for example, complement factor proteins (C2 [Q0V7N2], C4 [P01030] and C9 [A0A3Q1MU98]), coagulation factor V (F1N0I3; F5) and mannose-binding lectin protein (O02659; MBL). The unique proteins in HF cattle with reduced abundance were coagulation factor IX (F1MBC5; F9), keratin-2 (G3MZ71; KRT2) and Serpin A3-3 (G3N1U4; SERPINA3-3). Of 21 uniquely abundant proteins in the LF cattle, 13 showed increased abundance after BF exposure, including kininogen-2 (P01045; KNG2), plasminogen (P06868: PLG) and complement component 8 (Q2KIH5; C8A). The proteins with lower abundance in LF cattle were monocyte differentiation antigen CD14 (A8DBT6; CD14), thrombospondin-1 (Q28178; THBS1) and lipopolysaccharide-binding protein (F1MNN7; LBP).

**Figure 4:**
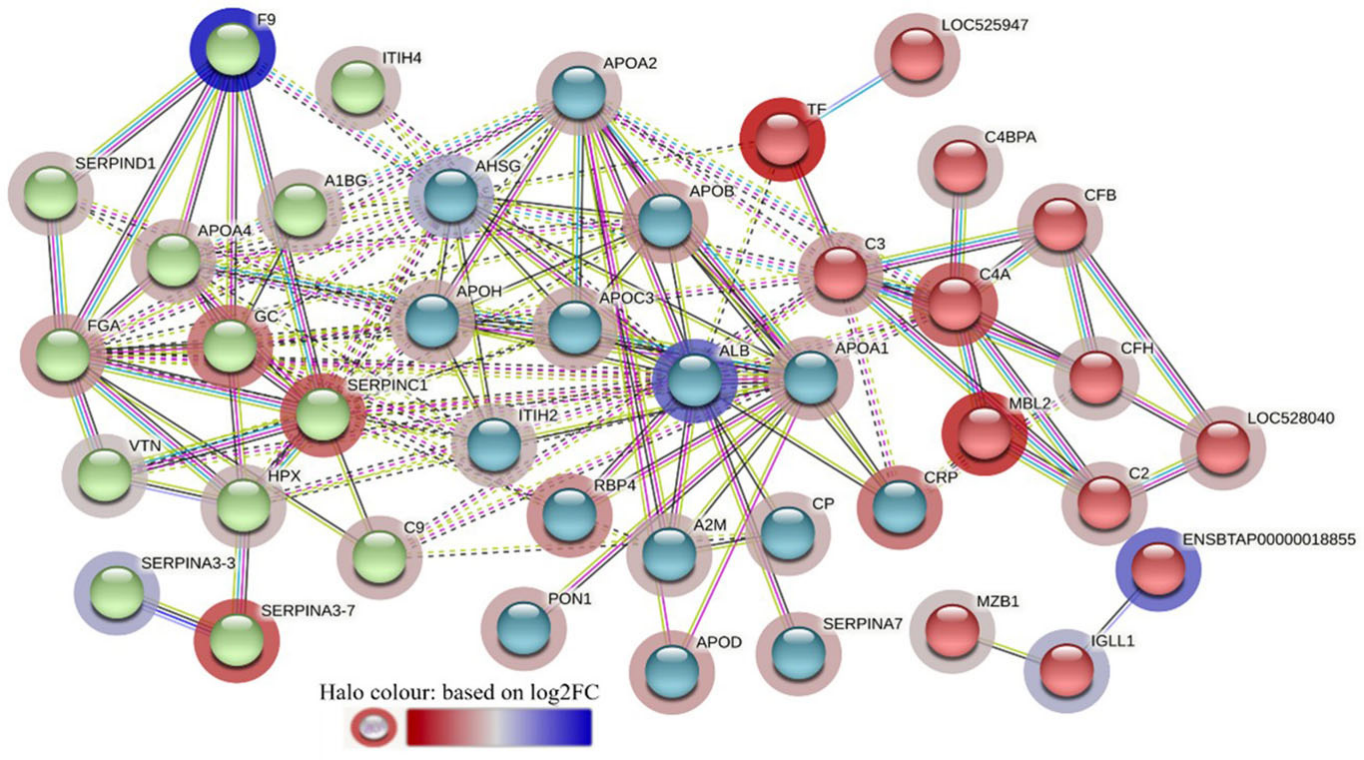
STRING protein interaction map based on biological process GO terms of differentially abundant proteins in high fly burden cattle in response to exposure to buffalo flies (HF-0 vs HF-1) with protein-protein interaction (PPI) p-value of < 1.0e-16. *K*-mean clusters showing strong interactions are highlighted as “red”, “green”, and “cyan blue” coloured nodes. Each node represents an individual protein and edges between nodes represent the predicted functional associations. Edges are drawn in evidence mode that uses 7 different line colours to indicate the type of interaction evidence: red indicates the presence of fusion evidence; green = neighbourhood evidence; blue = co-occurrence evidence; purple = experimental evidence; yellow = text-mining evidence; light blue = database evidence; and black = co-expression evidence. The solid and the dotted lines indicate connection within the same and different clusters, respectively. The halo colour is based on the log_2_FC value of the proteins in the dataset.

**Figure 5:**
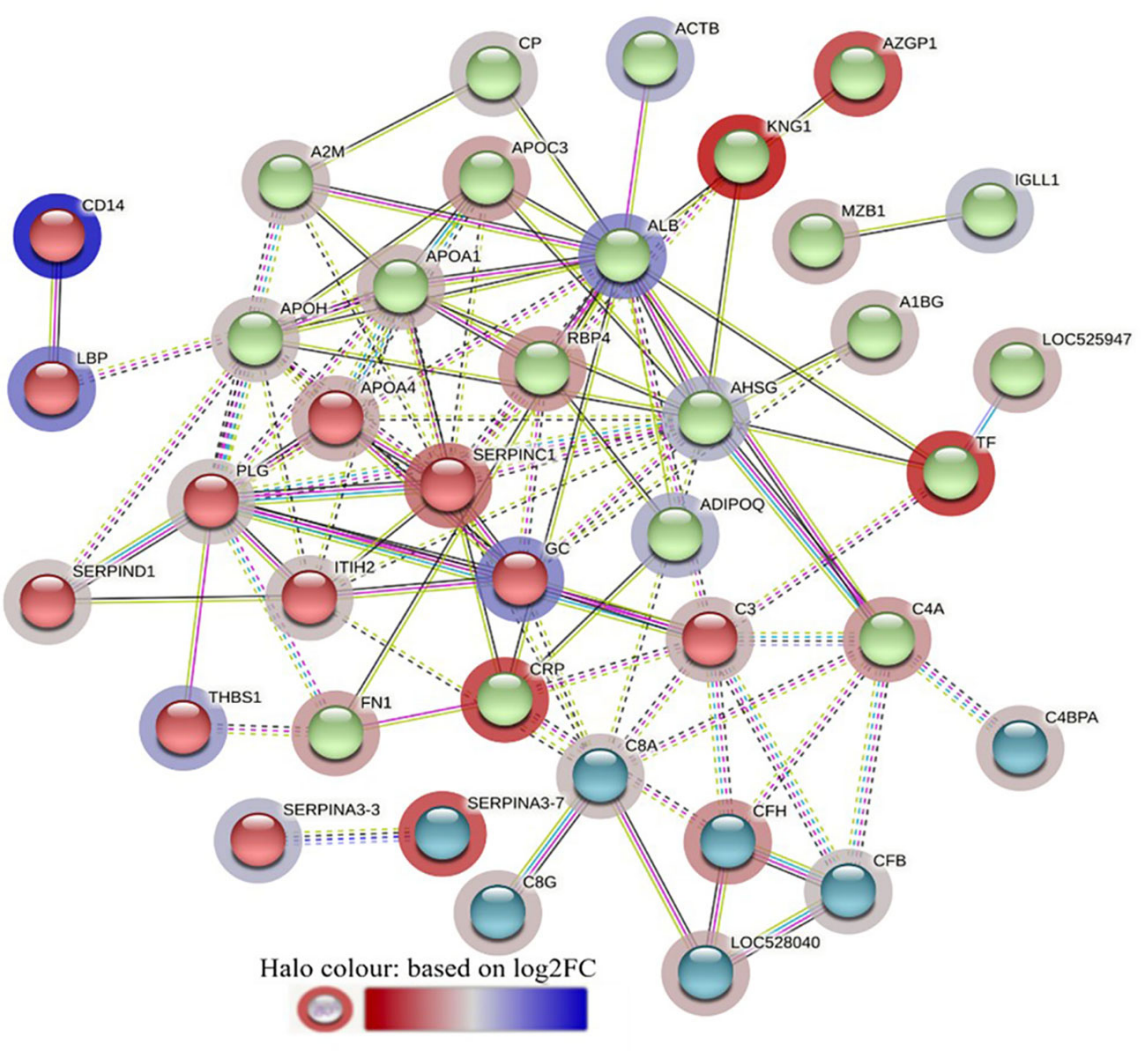
STRING protein interaction map based on biological process GO terms of differentially abundant proteins in LF-0 when compared to LF-1 cattle group with PPI p-value of < 1.0e-16. *K*-mean clusters showing strong interactions are highlighted as “red”, “green”, and “cyan blue” coloured nodes. Each node represents an individual protein and edges between nodes represent the predicted functional associations. Edges are drawn in evidence mode that uses 7 different line colours to indicate the type of interaction evidence: red indicates the presence of fusion evidence; green = neighbourhood evidence; blue = co-occurrence evidence; purple = experimental evidence; yellow = text-mining evidence; light blue = database evidence; and black = co-expression evidence. The solid and the dotted lines indicate connection within the same and different clusters, respectively. The halo colour is based on the log_2_FC value of the proteins in the dataset.

The STRING interaction analysis generated identical protein-protein interaction network maps for DA proteins in HF and LF cattle (**Figures 4**, **5**). The proteins in both groups were clustered into three distinct clusters, and the majority of proteins in these clusters were complement factors, apolipoproteins and serpin family members. The proteins in each of these clusters were slightly different in the two groups. For example, the complement factor cluster in HF cattle showed strong interactions between complement system proteins (C2, C3, C4A, CFB, CFH, C4BPA), LOC528040, MBL and TF in one cluster (**Figure 4**), whereas, in LF cattle, the complement cluster showed interactions between complement factors (CFB, CFH, C3, C4A, C4BPA, C8A, C8G), LOC528040 and SERPINA3-7 (**Figure 5**).

The GO term enrichment analysis identified that the DA proteins in both HF and LF cattle carried some common BP GO terms, for example, complement activation (GO:0006956), acute phase response (GO:0006953), blood coagulation (GO:0007596), response to wounding (GO:0009611), inflammatory responses (GO:0006954) and response to stress (GO:0006950) (**Supplementary File 1**: **Supplementary Tables 5.1**, **6.1**). The DA proteins in LF cattle were also enriched with some BP GO terms which were not present in HF cattle, for example wound healing (GO:0042060), regulation of phagocytosis (GO:0050764), immune system process (GO:0002376) and regulation of interleukin-8 production (GO:0032677). Regulation of proteolysis (GO:0030162) and negative regulation of lipase activity (GO:0060192) were among the unique BP GO terms in HF cattle. Complement and coagulation cascades (bta04610) KEGG pathway related to the host immune response were enriched in both high and low BF burden cattle groups after exposure to BFs involving 14 and 12 proteins, respectively (**Supplementary File 1**: **Supplementary Tables 5.2**, **6.2**). All the proteins involved in this pathway were at higher abundance in both cattle groups following BF exposure except CFH which was reduced in abundance in LF cattle. Additional BF GO terms and KEGG pathways are provided in **Supplementary File 1**: **Supplementary S5**, **S6**.

### 3.3 Comparison of serum proteomic profiles between high and low fly cattle

**Figure 6:**
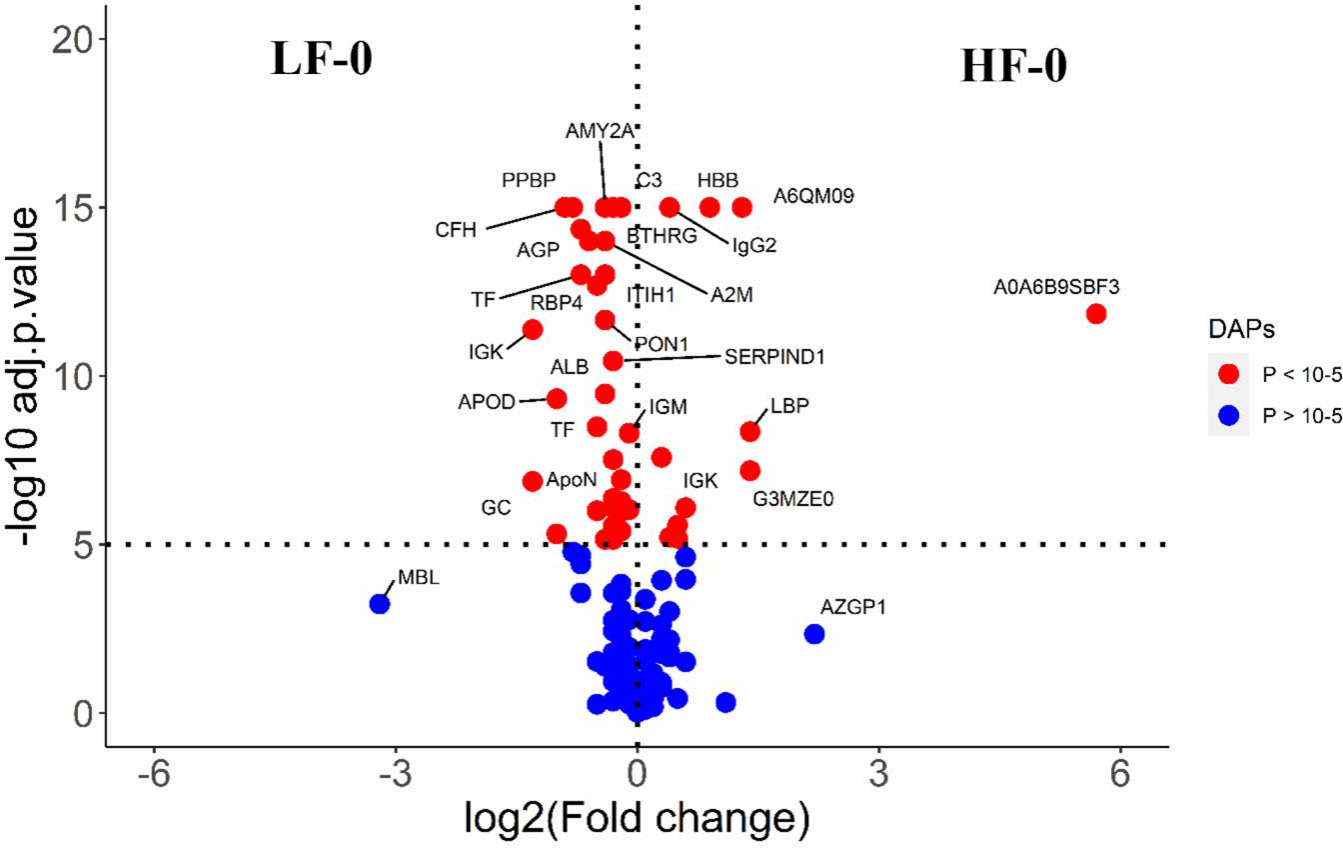
Volcano plot illustrating the DA proteins between HF-0 and LF-0 groups of cattle. Red, significantly different in abundance (P < 10-5). Blue, not significantly different in abundance (P > 10-5) using cut-off value of > 0.1 for log_2_ fold change (FC) as inclusion criteria of proteins. Positive scale of X-axis indicates HF-0 (naïve, high fly burden) cattle and negative scale represent LF-0 (naïve, low fly burden) cattle.

**Figure 7:**
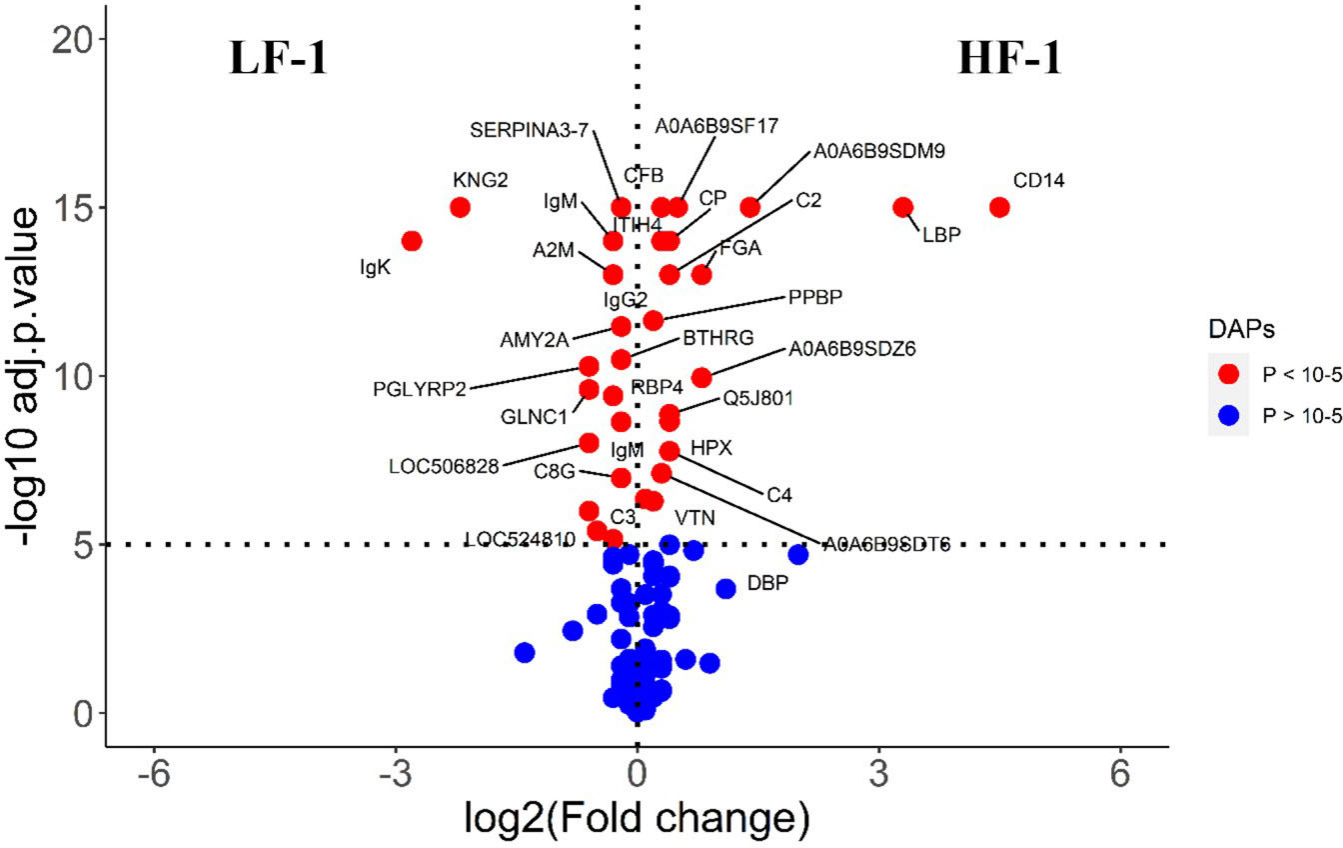
Volcano plot illustrating the DA proteins in high fly burden cattle post exposure to BFs when compared to low fly burden cattle. Red, significantly different in abundance (P < 10−5). Blue, not significantly different in abundance (P > 10−5) using cut-off value of > 0.1 for log_2_ fold change (FC) as inclusion criteria of proteins. Positive scale of X-axis indicates post exposure high buffalo fly burden cattle (HF-1) and negative scale represent post exposure low buffalo fly cattle (LF-1).

**Figure 8:**
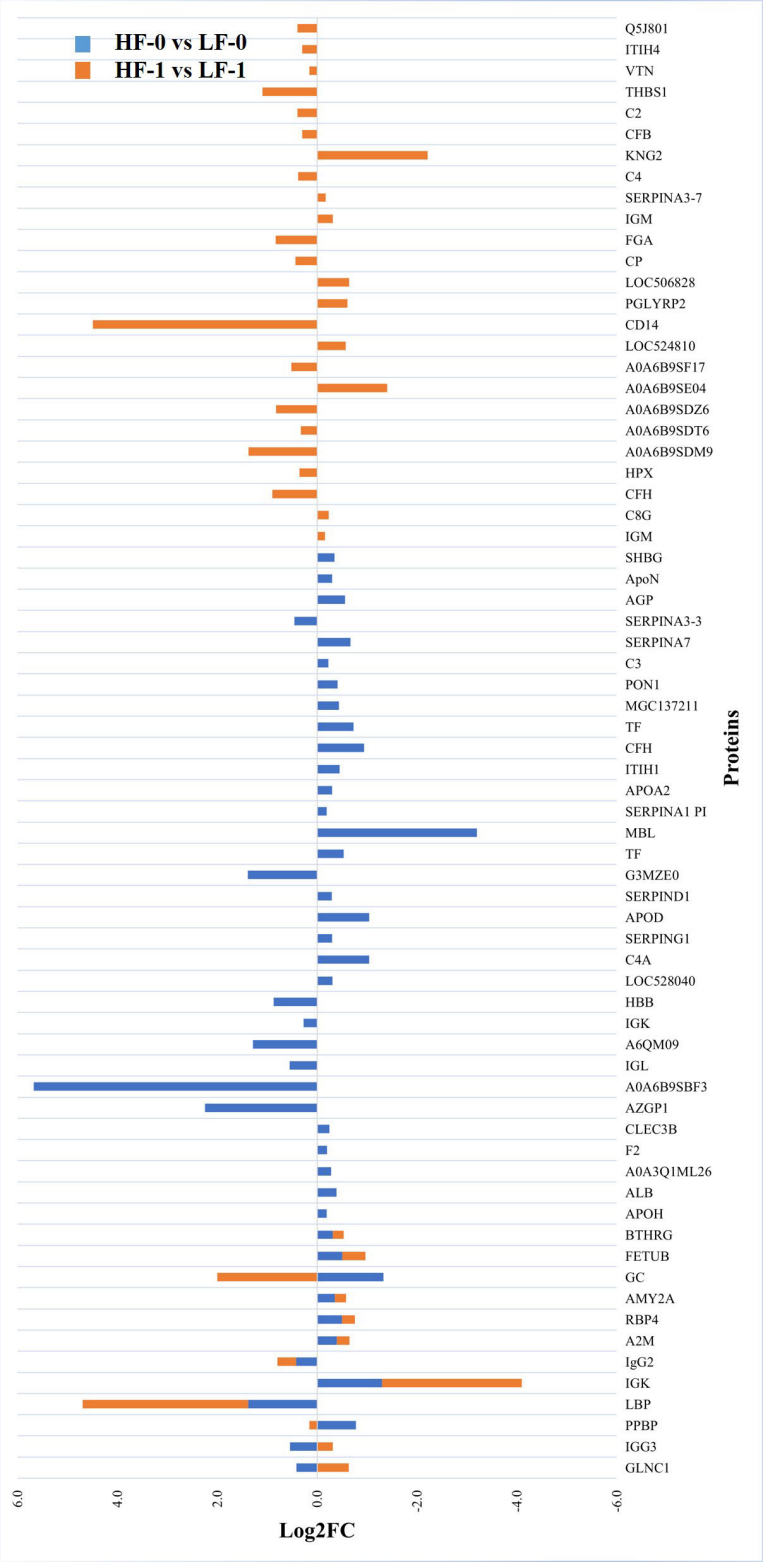
Relative abundance (log_2_ fold change (log_2_FC)) of significantly differentially abundant common and unique proteins between high fly and low fly burden naïve (HF-0 vs LF-0) indicated with blue colour bars and high fly and low fly burden post-exposure (HF-1 vs LF-1) cattle indicated with orange colour bars in response to BF exposure. Unique proteins in both comparisons are shown with single colour (either orange or blue) bars and common proteins are shown with double colour bars (orange and blue). A cut-off value of > 0.1 for log2 fold change (FC) is used as inclusion criteria of proteins.

The comparison of serum samples from the two naïve groups (HF-0 vs LF-0) identified 44 DA proteins with 32 proteins at higher abundance in LF cattle (**Figure 6**; **Supplementary File 1**: **Supplementary Table S7**). The HF-1 vs LF-1 comparison identified 37 proteins that were significantly different in abundance with 20 proteins significantly higher in the HF group as compared to the LF group (**Figure 7**; **Supplementary File 1**: **Supplementary Table S8**). There were 12 proteins common in both comparisons. Interestingly, four proteins showed a reciprocal pattern in abundance values; for example, immunoglobulin G3-like protein (A0A3Q1LPG0; IGG3) and globin C1 (A0A1K0FUD3; GLNC1) were higher in HF naïve cattle, but their abundance was higher in LF cattle after exposure. Similarly, C-X-C motif chemokine (F1MD83; PPBP) and Vitamin D-binding protein (Q3MHN5; GC) proteins were higher in LF naïve cattle, and their abundances were reduced when compared to HF cattle after exposure (**Figure 8**). Out of 12 common proteins, 2 proteins including lipopolysaccharide-binding protein (F1MNN7; LBP) and immunoglobulin gamma2 (G3N0V0; IgG2) were higher in HF-0 and HF-1 cattle. The other 6 proteins, out of 12 common proteins, were higher in abundance in both naïve and exposed LF cattle (**Figure 8**). The comparison of relative abundances of serum proteins between HF and LF cattle following BF exposure (HF-1 vs LF-1) provided an insight into the variation of serum proteomic profiles stimulated by BF infestation in both groups. The top most significantly abundant proteins in HF cattle in response to BF infestation included CD14, LBP and GC with log_2_FC 4.5, 3.3 and 2.0, respectively. In addition, HF cattle showed higher abundance of three immunoglobulin-like proteins (A0A6B9SDZ6, A0A6B9SF17 and A0A6B9SDT6) and four complement factor proteins (complement C2, C4, CFB and CFH). In the LF cattle, IGK, KNG2, and immunoglobulin heavy chain variable region (A0A6B9SE04) were among the top three significant DA proteins with log_2_FC 2.8, 2.2 and 1.4, respectively. The LF cattle also showed an increased abundance of some other immune-related proteins including immunoglobulin-M precursors (A5D7Q2 and G5E5T5), IgG3 heavy chain constant region (A0A3Q1LPG0) and complement C8 gamma chain (A0A3Q1ME55).

Most proteins that showed significantly higher abundance in HF naïve cattle were uncharacterised proteins which were identified as immunoglobulin-like proteins following a BLAST search. For example, some of the highly abundant proteins were immunoglobulin heavy chain variable region (A0A6B9SBF3), AZGP1 and immunoglobulin-like domain containing protein (G3MZE0) with log_2_FC values of 5.7, 2.2 and 1.4, respectively. The most highly abundant proteins in LF cattle were MBL, GC and immunoglobulin K protein (IGK; F1MZ96) with log_2_FC values of 3.2, 1.3 and 1.3, respectively. In addition, the abundance of three complement factors, including CFH, complement C3 isoform X1 (E1B805) and C3 was also significantly higher in LF naïve cattle compared to HF naïve group.

**Figure 9:**
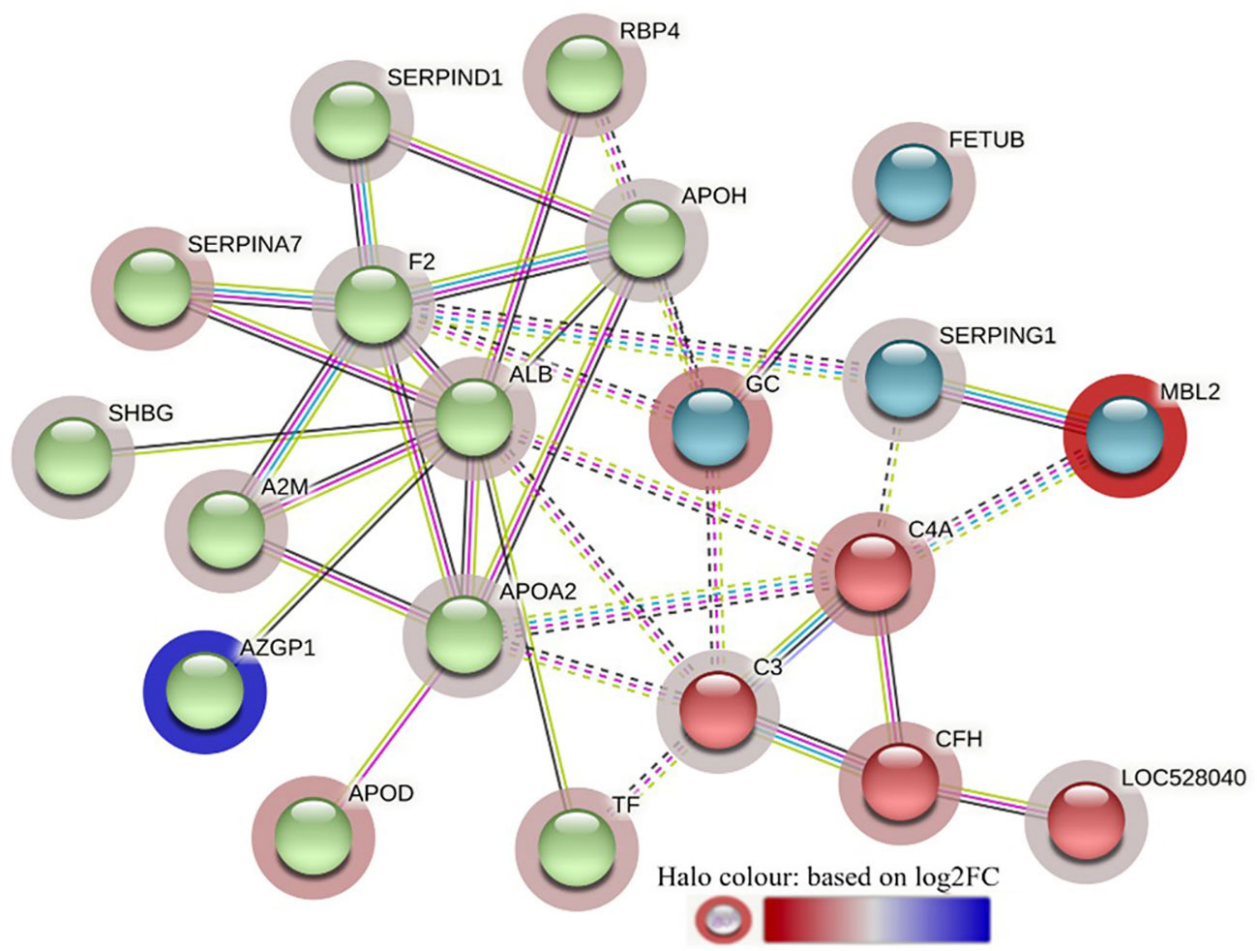
STRING protein interaction map based on biological process GO terms of differentially abundant proteins in HF-0 (naïve, high fly burden) cattle when compared to LF-0 (naïve, low fly burden) cattle with PPI p-value of < 1.0e-16. *K*-mean clusters showing strong interactions are highlighted as “red”, “green”, and “cyan blue” coloured nodes. Each node represents an individual protein and edges between nodes represent the predicted functional associations. Edges are drawn in evidence mode that uses 7 different line colours to indicate the type of interaction evidence: red indicates the presence of fusion evidence; green = neighbourhood evidence; blue = co-occurrence evidence; purple = experimental evidence; yellow = text-mining evidence; light blue = database evidence; and black = co-expression evidence. The solid and the dotted lines indicate connection within the same and different clusters, respectively. The halo colour is based on the log_2_FC value of the proteins in the dataset.

The STRING analysis of the DA proteins from HF-0 vs LF-0 comparison generated a protein-protein interaction map shown in **Figure 9**. Based on *k-*mean clustering, four complement proteins (C3, C4A, CFH and C3 isoform X1) were grouped together in one cluster. The apolipoproteins (APOD and APOH), serpin family members (SERPIN D-1 and SERPIN A-7), RBP4, prothrombin (F2), ALB, TF, AZGP1, sex hormone binding globulin (SHBG) and A2M were grouped together in another cluster. In this cluster, the only protein at higher abundance in the HF cattle (HF-0) was AZGP1. The third cluster was composed of MBL and SERPING1. Gene ontology enrichment analysis showed that most of the proteins (for example, C3, C4A, CFH, MBL) with higher abundance in LF cattle were involved in the activation of the complement system (GO:0006956), including alternative (GO:0006957) and classical pathways (GO:0006958) and immune response related biological processes including immunoglobulin mediated immune response (GO:0016064), blood coagulation (GO:0007596), response to wounding (GO:0009611) and inflammatory response (GO:0006954) (**Supplementary File 1**: **Supplementary Table 9.1**). The KEGG pathway analysis showed that DA proteins with higher abundance in LF cattle were enriched in complement and coagulation cascades (bta04610) (**Supplementary File 1**: **Supplementary Table 9.2**).

**Figure 10:**
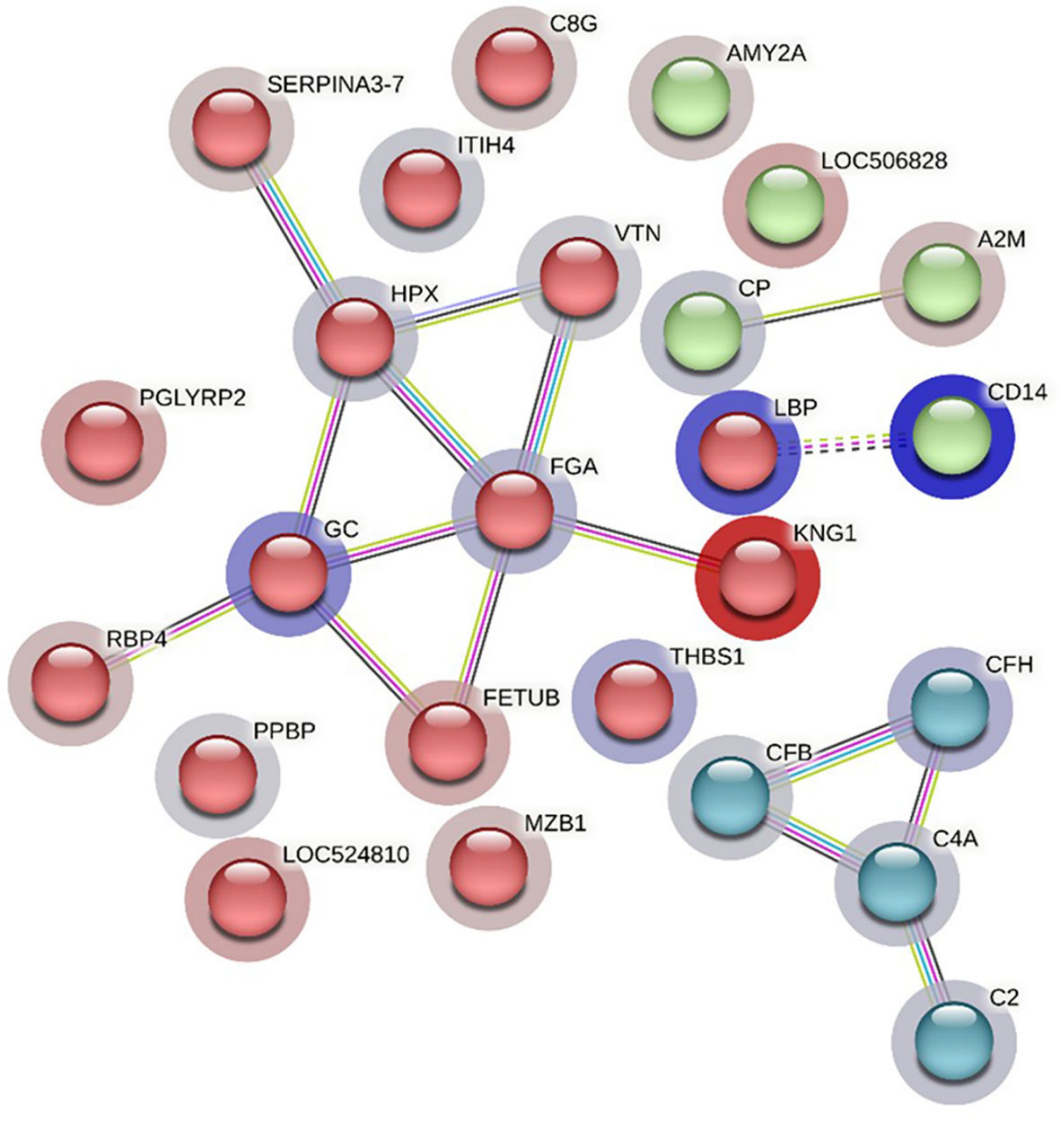
STRING protein interaction map based on biological process GO terms of differentially abundant proteins in HF-1 (high fly burden) cattle when compared to LF-1 (low fly burden) cattle after exposure to BFs with PPI p-value of < 1.0e-16. *K*-mean clusters showing strong interactions are highlighted as “red”, “green”, and “cyan blue” coloured nodes. Each node represents an individual protein and edges between nodes represent the predicted functional associations. Edges are drawn in evidence mode that uses 7 different line colours to indicate the type of interaction evidence: red indicates the presence of fusion evidence; green = neighbourhood evidence; blue = co-occurrence evidence; purple = experimental evidence; yellow = text-mining evidence; light blue = database evidence; and black = co-expression evidence. The solid and the dotted lines indicate connection within the same and different clusters, respectively. The halo colour is based on the log_2_FC value of the proteins in the dataset.

The STRING analysis of differentially abundant proteins in the HF-1 vs LF-1 comparison showed a strong interaction between the complement proteins (C2, C4A, CFH and CFB) in the same *k*-mean cluster and were associated with complement activation (GO:0006956). The second k-mean cluster showed an interaction between proteins that are associated with blood coagulation, including VTN, HPX, fibrinogen alpha chain (FGA; F6QND5), serpin A3-7 (SERPINA3-7; G8JKW7), KNG, and fetuin-B (FETUB; Q58D62). Monocyte differentiation antigen CD14 and LBP were connected in a separate cluster. The GO term enrichment analysis showed that DA proteins in this comparison had significant enrichment in humoral immune response (GO:0006959), inflammatory response (GO:0006954) and defence response (GO:0006952), with the majority of the proteins involved in these biological processes at higher abundance in HF cattle following BF exposure (**Figure 10**; **Supplementary File 1**: **Supplementary Table 10.1**). The KEGG pathway analysis showed that DA proteins were enriched in NF-kappa B signalling pathway (bta04064), phagosome (bta04145) and complement and coagulation cascades (bta04610) (**Supplementary File 1**: **Supplementary Table 10.2**).

## 4 Discussion

Direct phenotyping of cattle for BF and horn fly numbers is laborious and often inaccurate. The availability of immune or genomic biomarkers for identifying BF-resistant cattle could facilitate practical selection and breeding, but very little is known about the immune responses of cattle to BFs and how the host regulates fly burden. Technologies for identifying biomarkers, including genomics, transcriptomics and proteomics can potentially be used in cattle breeding programs and this field has greatly advanced in recent years. The use of proteomic techniques has increased markedly as they have the potential to greatly assist in disease diagnostics and the discovery of biomarkers for host resistance to pathogens and parasites (21, 27–29). For clinical proteomics research, the most common biological materials used are blood, serum or plasma. These are easily obtained and contain measurable protein biomarkers that can reveal physiological and disease-related changes (30). This study used serum proteomics to identify bovine biomarkers associated with low buffalo fly numbers in *Bos indicus x Bos taurus* (Brangus) composite breed cattle.

Bovine host responses to BFs could be of interest in breeding cattle for resistance in two main ways. Firstly, the immune response could have a regulatory effect on BFs by affecting the efficiency of BF feeding and reproduction. Secondly, the magnitude or nature of the immune response could be correlated with BF burden and provide a potential indirect indicator of BF susceptibility. In this study, the differences in serum proteomes of cattle with high and low BF burden were investigated using SWATH-MS to explore host response to BFs and measure the differences in serum proteomic profiles of cattle in the two groups.

Within-group comparisons provided information about the responses of HF and LF cattle to BF infestation by comparing the post-exposure samples with samples collected from naïve cattle. Buffalo fly infestation evoked a similar response in HF and LF cattle; for example, 48 DA proteins were common to both groups, and these proteins were associated with immune response-related processes such as complement activation, humoral and leukocyte-mediated immunity, blood coagulation and inflammatory response. Anticoagulatory mechanisms such as fibrinolysis that may facilitate blood-feeding were prominent in HF cattle following BF infestation along with reduced abundance of coagulation factor IX. Recently the abundance of proteins involved in fibrinolysis in response to tick infestation was reported as higher in tick susceptible, but not in tick resistant cattle (31). However, naïve tick resistant cattle had higher abundance of carboxypeptidase B2 (thrombin-activatable fibrinolysis inhibitor) than the tick susceptible cattle. Many hematophagous ectoparasites, including ticks, have the ability to stimulate fibrinolysis *in vitro* (32) and these mechanisms also contribute to the maintenance of blood flow and facilitate feeding by these parasites.

In addition, the abundance of coagulation factor IX (F9), was decreased in the HF group following BF infestation. Coagulation factor 9 is an important protein in haemostasis and plays a key role in the coagulation cascade (33). Decreased relative abundance of F9 after BF exposure suggests that BF salivary components may be inhibiting blood coagulation and facilitating blood feeding. In contrast, pro-coagulation processes, including negative regulation of fibrinolysis and regulation of plasminogen activation were significantly higher in LF cattle.

Ribeiro (34) noted that arthropods that feed directly from capillaries, such as mosquitoes (solenophages), generally have a sophisticated array of anti-haemostatic molecules in their saliva to overcome a wide range of haemostatic mechanisms that may include vasoconstriction and platelet aggregation in addition to blood coagulation. In comparison, pool feeding arthropods (telmophages) such as BF and horn flies generally have much less complex saliva that frequently targets thrombin or factors involved in the thrombin pathway. Kerlin and Hughes (35) compared the concentration of apyrase, an enzyme that inhibits ADP-dependent platelet aggregation, in salivary gland homogenates from hematophagous arthropods including mosquitoes (*Aedes aegypti*), ticks (*Rhipicephalus australis*) and BF. The concentration of apyrase was high in mosquitoes that feed from capillaries where any clotting or aggregation of platelets would severely compromise feeding efficiency. However, although apyrase was present in ticks, the levels were lower. Cattle ticks feed from a pool of blood formed beneath their mouth­parts which are anchored in the dermis and may require only small amounts of apyrase to maintain permissive levels of platelet aggregation. With BFs, which abrade the skin and feed from the resultant pooled blood, then rapidly move to a new feeding site, the concentration of apyrase was near the limit of detection and much lower than for both mosquitoes and ticks. Even though horn fly adults feed recurrently on their hosts, these flies lack the ADP-responsive antiplatelet aggregation and vasodilatory anti-haemostatic systems described for other blood-feeding arthropods (36).

It was also noted by Untalan et al. (37) that the composition of horn fly saliva was relatively simple in comparison to capillary-feeding ectoparasites, with only one anti-haemostatic factor, thrombostasin, identified in horn fly saliva at the time of their report. Vaccination against recombinant thrombostasin reduced blood meal size and delayed horn fly egg development (38). More recently, at least two more immunomodulatory compounds that may regulate horn fly numbers (haematobin and irritans 5) have been identified (16). It has been shown that haematobin modulates macrophage inflammatory response, whereas irritans 5 was hypothesised to have immunoglobin binding properties (39).

Moreover, other proteins also associated with blood coagulation and wound healing (F2, APOH, APOD, SERPING1, SERPIND1) showed a higher abundance in naïve LF cattle in our study, further indicating that active blood coagulation processes may provide an unsuitable environment for BF feeding in more resistant cattle. Serpins also play an important role in regulating blood coagulation and complement activation pathways. For example, *R. australis* serpins transcribed during feeding appear to regulate tick physiology and interactions with the host immune system (40).

Thrombin has been implicated in resistance of cattle to horn files in several instances. It has been suggested that variations between breeds of cattle and individuals within breeds in thrombin genotype may underlie differences in cattle in susceptibility to horn flies (37, 41) and in a recent study, blood thrombin levels were shown to be negatively correlated with horn fly burden in cattle, although the association was relatively weak (r= -0.13) (38). Horn flies are very closely related to BFs and we have shown that BF also secrete thrombostasin in their saliva (unpublished observations). Our results provide evidence that differences in the strength of the coagulation response may also be a key factor underlying variation amongst cattle in susceptibility to BF and suggest that further study of proteins related to the coagulation cascade and immune response could provide practical biomarkers or genomic indices for breeding increased resistance to *Haematobia* spp. flies.

Our findings show that although both groups of cattle developed an immune response to BF infestation, the response of LF cattle was generally stronger. The abundance of immunoglobulin-like protein was increased by log_2_FC value of 5.5 in response to BF infestation in LF cattle, whereas the highest log_2_FC value for immunoglobulins like proteins in HF cattle was 1.0. In our findings, LF cattle also efficiently modulated other mechanisms such as phagocytosis and wound healing. Modulation of immune response may have also contributed to reducing favourability for BF feeding. Similar responses have also been reported to ticks in cattle where it was found that although infestation elicited similar responses in resistant and susceptible cattle, the immune responses were stronger in the resistant animals and tick-susceptible cattle showed higher abundance of proteins that facilitated tick-feeding (21, 31).

Previously, Kerlin and Allingham (17) reported that cattle exposed to BFs developed serum antibodies to BF antigens at levels correlating with the intensity of exposure, however, these antibodies were not protective. Thus, higher levels of immune proteins in HF cattle following infestation may simply reflect a greater antigen challenge in these cattle. The abundance of proteins involved in response to stress was also increased in HF cattle, which is likely due to the effects of greater BF burden. For example, thrombospondin showed a higher abundance in HF cattle following exposure to BFs. Thrombospondin has been shown to increase in response to acute stress conditions (42) and overexpression leads to endoplasmic reticulum stress that promotes apoptosis and inflammation (43).

The comparisons between groups within time (HF-0 vs LF-0 and HF-1 vs LF-1) also provided information about variations in serum proteomic profiles that may contribute to differences amongst cattle in BF numbers. Naïve LF cattle showed higher abundance of proteins associated with innate immune response when compared to naïve HF cattle. Most of the DA proteins were involved in complement activation (C3, C4A, CFH, MBL) and immunoglobulin-mediated immune response. In addition, the abundance of MBL was higher in LF cattle than HF cattle, both before and after BF exposure and this suggests an ability of these cattle to develop a rapid and efficient response against BFs. Mannose-binding lectin has been suggested to play a crucial role in the first hours/days of primary immune responses (44), thus providing the host with a first line of defence until the adaptive immune responses develop. Mannose-binding lectin has previously been associated with the regulation of host resistance to protozoan parasites and MBL-deficient mice had a higher *Trypanosoma cruzi* burden in blood and tissue compared to normal control animals (45). Similarly, Antony et al. (46) reported that MBL is associated with protection against *Schistosoma japonicum* through its interaction with the tegument of *S. japonicum*, which results in the activation of complement cascades. Mannose-binding lectin has also been suggested as a potential biomarker in the sera of rabbits infected with *S. japonicum* (47).

In addition, the abundance of C-X-C motif chemokine was higher in naïve LF than naïve HF cattle, but the abundance was higher in HF cattle following BF exposure. C-X-C motif chemokine acts in inflammatory response by attracting neutrophils and other leukocytes to the site of inflammation (48), and the increased abundance of this protein in HF cattle after BF exposure may indicate elevated levels of cutaneous inflammation in response to high fly burdens. This was also suggested by the significant enrichment of the inflammatory process GO term. Local inflammation promotes blood circulation, and this could similarly act to increase accessibility to blood and the numbers of BF found on HF cattle (49). Breijo et al. (50) indicated that vaccinating against haematobin, an immunomodulator identified in HF saliva, could downregulate the production of inflammatory mediators by macrophages, thereby reducing the favourability of host skin for HF feeding and reducing HF numbers on cattle by 30%.

The concept of breeding for disease resistance through selecting for general immune competence, also referred to as immune resilience, has recently been advanced with reference to Angus cattle (51). It was suggested that this could be accomplished by selecting animals with an enhanced ability to mount both antibody and cell mediated immune responses and selection for immune responsiveness has been successfully used to reduce the incidence of disease in both swine and dairy cattle (52, 53). In a more recent paper with sheep, evidence was provided that selection for immune resilience may also provide benefits in reducing the effects of parasitic disease (54). The higher abundance of immune-related proteins in naïve LF cattle observed in our study could also reflect the effects of generalised disease resilience in modulating BF numbers.

## 5 Conclusions

The findings of this study showed that although LF and HF cattle developed very similar responses to BF infestation, certain proteins associated with anti-coagulation and pro-inflammatory processes such as antithrombin III, C4a and C-X-C motif chemokine were higher in HF cattle. In comparison more resistant (LF) cattle maintained the mechanisms that promote blood coagulation, thus reducing the blood supply for BF feeding. In addition, naïve LF cattle had stronger innate immune-response mechanisms than HF cattle, which may enable them to develop an early protective response against BFs. These results suggest that underlying differences in the abundance of proteins related to blood coagulation and immune response pathways could potentially provide indirect indicators of BF burden and biomarkers for selecting more BF-resistant cattle. This will require validation in studies with a wider range of cattle genotypes and under varying management and environmental conditions.

## Data availability statement

The datasets presented in this study can be found in online repositories. The names of the repository/repositories and accession number(s) can be found below: PXD039655 (ProteomeXchange).

## Ethics statement

The animal study was approved by The University of Queensland Animal Ethics Committee. The study was conducted in accordance with the local legislation and institutional requirement (Certificate number QAAFI/469/18).

## Author contributions

MK: Conceptualization, Data curation, Formal analysis, Investigation, Methodology, Software, Writing – original draft, Writing – review & editing. AR: Conceptualization, Data curation, Formal analysis, Investigation, Methodology, Project administration, Software, Supervision, Validation, Visualization, Writing – original draft, Writing – review & editing. MN: Investigation, Writing – review & editing. CT: Supervision, Writing – review & editing. AT: Conceptualization, Funding acquisition, Project administration, Resources, Supervision, Writing – review & editing. PJ: Conceptualization, Data curation, Formal analysis, Funding acquisition, Investigation, Project administration, Resources, Supervision, Validation, Writing – review & editing.
